# Added value of Gd-EOB-DTPA-enhanced Hepatobiliary phase MR imaging in evaluation of focal solid hepatic lesions

**DOI:** 10.1186/1471-2342-13-41

**Published:** 2013-12-01

**Authors:** Michael Haimerl, Max Wächtler, Ivan Platzek, Rene Müller-Wille, Christoph Niessen, Patrick Hoffstetter, Andreas Georg Schreyer, Christian Stroszczynski, Philipp Wiggermann

**Affiliations:** 1 Department of Radiology, University Medical Center Regensburg, Regensburg 93042, Germany; 2 Department of Radiology, University Hospital Carl Gustav Carus Dresden, Dresden, Germany

**Keywords:** Magnetic resonance imaging, Focal hepatic lesions, Gd-EOB-DTPA-enhanced MR imaging, Hepatobiliary, Diagnostic performance, Characterization

## Abstract

**Background:**

Correct characterization of focal solid hepatic lesions has always been a challenge and is of great diagnostic and therapeutic relevance. The purpose of this study was to determine the added value of hepatobiliary phase images in Gd-EOB-DTPA-enhanced magnetic resonance imaging (MRI) for differentiating focal solid hepatic lesions.

**Methods:**

In this retrospective trial 84 consecutive patients underwent Gd-EOB-DTPA-enhanced MR examinations. MRI was conducted for 64 patients with malignant focal hepatic lesions (34 hepatocellular carcinoma (HCC), 30 metastases) and for 20 patients with benign hepatic lesions (14 focal nodular hyperplasia (FNH), 3 adenoma, 3 hemangioma). Five radiologists independently reviewed three sets of MR images by means of a 5-point confidence scale from score 1 (definitely benign) to score 5 (definitely malignant): set 1: unenhanced images; set 2: unenhanced and Gd-EOB-DTPA-enhanced dynamic images; set 3: hepatobiliary phase images in addition to set 2. Accuracy was assessed by the alternative free-response receiver operating characteristic curve (A_z_) and the index of diagnostic performance was calculated.

**Results:**

Diagnostic accuracy was significantly improved by the addition of Gd-EOB-DTPA-enhanced dynamic images: A_z_ in set 1 was 0.708 and 0.833 in set 2 (P = 0.0002). The addition of hepatobiliary phase images increased the A_z_ value to 0.941 in set 3 (set 3 vs set 2, P < 0.0001; set 3 vs set 1, P < 0.0001). The index of diagnostic performance was lowest in set 1 (45%), improved in set 2 (71%), and highest in set 3 (94%).

**Conclusions:**

Hepatobiliary phase images obtained after Gd-EOB-DTPA-enhanced dynamic MRI improve the differentiation of focal solid hepatic lesions.

## Background

Focal solid hepatic lesions include primary liver malignancies, metastases, and benign tumor-like lesions. The detection and accurate characterization of such lesions is of high clinical importance for surgical interventions and minimally invasive tumor therapies. MR imaging (MRI) of the liver with an extracellular contrast medium, such as gadopentetate dimeglumine (Gd-DTPA) and particularly liver-specific contrast agents, has been shown to increase the detection rate and the diagnostic accuracy of focal solid hepatic lesions in comparison to other noninvasive modalities including ultrasonography (US) and computed tomography (CT) [1-4]. Over the past 10 years, several liver-specific MRI contrast media have been developed, for example, superparamagnetic iron oxide particles targeting Kupffer cells and gadobenate dimeglumine (Gd-BOPTA) or mangafodipir trisodium (Mn-DPDP), which are taken up by hepatocytes. These contrast agents have been investigated in clinical trials with the objective of increasing the performance of MR liver imaging [5-7]. Recently, the liver-specific hepatobiliary contrast agent Gadolinium ethoxybenzyl diethylenetriaminepentaacetic acid (Gd-EOB-DTPA, gadoxetic acid disodium, Primovist, Schering, Berlin, Germany) has been introduced for hepatic MRI examinations. Gd-EOB-DTPA is a hydrophilic, paramagnetic, highly water-soluble, and therefore bolus-injectable Gd-DTPA derivate for T1-weighted MRI that allows the evaluation of delayed hepatocyte uptake and biliary excretion [8,9]. Approximately 50% of the injected dose is taken up by functioning hepatocytes and excreted in bile compared to a hepatocellular uptake rate of 3% to 5% for gadobenate dimeglumine [10].

In principle, injected Gd-EOB-DTPA accumulates in hepatocytes during the hepatobiliary phase. Consecutive normal areas of the liver ─ in contrast to malignant focal hepatic lesions, such as metastases ─ exhibit T1-shortening. Gd-EOB-DTPA produces both dynamic perfusion and liver-specific hepatobiliary MR images, thus combining the properties of an extracellular fluid contrast agent and a hepatobiliary agent [11,12].

Recent studies showed that Gd-EOB-DTPA-enhanced hepatobiliary phase MR images can help differentiating hepatocellular carcinoma (HCC) from arterial enhancing pseudolesions and that combined reading with precontrast and Gd-EOB-DTPA-enhanced MR images may improve the diagnostic performance for the diagnosis of HCCs [13-15].

The purpose of this trial was to determine the value of hepatobiliary phase post-contrast images in gadoxetic acid-enhanced MRI in addition to unenhanced and contrast-enhanced dynamic MRI for obtaining the highest possible accuracy in characterizing such lesions in the clinical routine of a university hospital.

## Methods

### Patients

**Figure 1:**
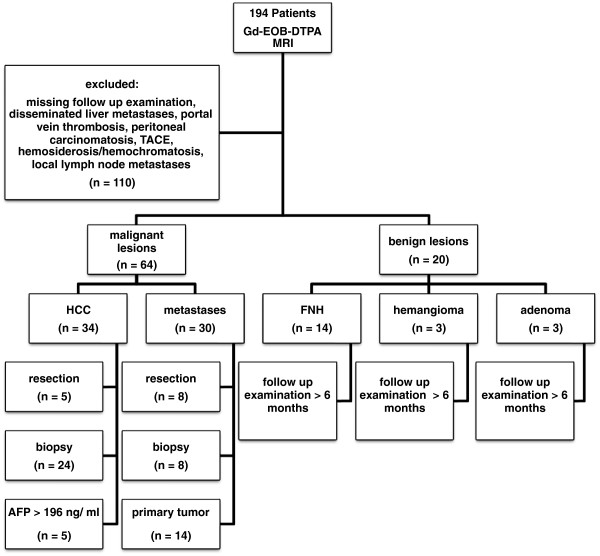
**Flowchart of patients and lesions included in this trial.** 194 patients underwent Gd-EOB-DTPA-enhanced MRI of the liver. 110 patients were excluded, mainly because of missing follow-up examinations, and 84 consecutive patients were included. Malignant lesions (n = 64) were proven by histology (n = 45), or diagnosis was either based on AFP > 196 ng/ml (n = 5) or knowledge of the primary tumor in case of metastases (n = 14). Benign lesions (n = 20) showed no change on follow-up examinations for more than 6 months.

The Ethics Committee deemed approval of this retrospective trial unnecessary. Between January 2009 and September 2010, 194 consecutive patients with focal hepatic lesions diagnosed using US or CT examination underwent hepatic gadoxetic acid disodium-enhanced MRI to confirm or rule out malignancy. Exclusion criteria were portal vein thrombosis, as well as obvious malignancies, such as disseminated liver metastases, peritoneal carcinomatosis, or local lymph node metastases. Other exclusion criteria were history of chemotherapy including transcatheter arterial chemoembolization (TACE), history of thermal ablation, presence of hemosiderosis or hemochromatosis. Eighty-four patients, i.e. 54 men (mean age: 66.7; age range: 43 to 86 years) and 30 women (mean age: 53.2; age range: 21 to 83 years), met the inclusion criteria of this trial. Figure 1 summarizes the patient flowchart.

### Standard of reference

Diagnosis of HCC was either confirmed by histology, based on surgical findings (n = 5) or on results of percutaneous biopsies (n = 24), or obtained from pathologically elevated α-fetoprotein (AFP) values (>196 ng/ml) (n = 5). The diameter of the 34 HCCs ranged from 0.8 cm to 14 cm. Diagnosis of hepatic metastases was based on resection (n = 8), percutaneous biopsy (n = 8), or typical image analysis if the primary tumor was known (n = 14). Twenty lesions were considered benign (focal nodular hyperplasia (FNH) n = 14, hemangioma n = 3, adenoma n = 3) because of the absence of interval changes in the follow-up CT or MR images for more than 6 months without treatment. Figure 1 summarizes how diagnosis of hepatic lesions was confirmed.

### MR Imaging

Gadoxetic acid-enhanced MRI was conducted for all patients by means of a 1.5-T system (Magnetom Symphony, Siemens, Erlangen, Germany) with the manufacturer’s body and spine array coils. The entire liver was imaged in the transverse plane.

First, we obtained respiratory-triggered single-shot T2-weighted turbo spin-echo images (repetition time (TR)/echo time (TE): 1000/85; flip angle: 150°; slice thickness: 6 mm; matrix: 180 × 320) followed by two different breath-hold fast-spoiled gradient-echo images, i.e. T1-weighted in-phase (repetition time (TR)/echo time (TE): 87/4.8; flip angle: 60°; slice thickness: 6 mm; matrix: 154 × 320) and T1-weighted out-of-phase images (repetition time (TR)/echo time (TE): 100/2.7; flip angle: 70°; slice thickness: 6 mm; matrix: 154 × 320).

Then, we conducted a three-dimensional T1-weighted gradient-echo sequence (repetition time (TR)/echo time (TE): 4.0/1.5; flip angle: 10°; slice thickness: 6 mm; matrix: 174 × 320) using the fat suppression technique. After the administration of 10 ml Gd-EOB-DTPA (Primovist; Bayer Schering, Berlin, Germany), the same sequence was repeated with dynamic contrast-enhanced MRI. The contrast material was injected with a power injector as a bolus at a rate of 2 mL/s via a 22-gauge intravenous cubital line flushed with 15 mL of saline. Dynamic contrast-enhanced MRI was initiated after the start of the bolus injection to obtain multi-phasic images (arterial, portal, and equilibrium phases). Additionally, hepatobiliary phase images were obtained after 20 min using the same dynamic contrast-enhanced MR sequence. Also, respiratory-triggered T2-weighted turbo spin-echo images with fat suppression (repetition time (TR)/echo time (TE): 2220/79; flip angle: 140°; slice thickness: 6 mm; matrix: 320 × 320) and respiratory-triggered diffusion-weighted images (repetition time (TR)/echo time (TE): 1900/72; slice thickness: 6 mm; matrix: 144 × 192) were obtained between the late phase and the hepatobiliary phase as part of the routine liver MRI protocol (Table 1).

**Table 1:** MR imaging parameter

**Parameter**	**T2-weighted imaging unenhanced**	**T1-weighted imaging unenhanced**	**Contrast-enhanced imaging**
Sequence	Fast spin-echo	Gradient-echo, FLASH	Gradient-echo, FLASH
Respiratory-triggered	Yes	No	No
Matrix	180 × 320	174 × 320	174 × 320
Section thickness (mm)	6	6	6
Flip angle (degrees)	150	10	10
Field of view	285 × 380	344 × 380	344 × 380
Repetition time (ms)	1000	4.0	4.0
Echo time (ms)	85	1.5	1.5
Acquisition time	>79 s	17 s	17 s

### Imaging analysis

Five radiologists (with 1, 5, 6, 10, and 16 years of experience in abdominal imaging respectively) independently reviewed 3 sets of MR images in random order: set 1, unenhanced (precontrast T1- and T2-weighted) images; set 2, unenhanced and gadoxetic acid-enhanced dynamic images (arterial, portal, and equilibrium phases); set 3, unenhanced, gadoxetic acid-enhanced dynamic images (arterial, portal, and equilibrium phases) and hepatobiliary phase images 20 min after Gd-EOB-DTPA bolus injection. The 5 observers were blinded to laboratory results, patient histories, findings of other imaging modalities, and final diagnoses. The interval between the reviews of the 3 sets of images was at least 3 weeks.

Each observer used a 5-point confidence rating scale for the respective set to evaluate each lesion as follows: score 1: definitely benign, score 2: preferentially benign, score 3: unclear; score 4: preferentially malignant; score 5: definitely malignant.

### Statistical analysis

Statistical analyses were done with a commercially available software program (MedCalc, version 12.1.4, MedCalc Software, Mariakerke, Belgium). The means of age between men and women were compared with the Mann–Whitney U-test. A receiver operating characteristic analysis with the maximum likelihood estimation program (ROCKIT DBMMRMC 2.2 B3, C.E. Metz, University of Chicago, Chicago, Ill) was conducted to show the diagnostic performance with dynamic gadoxetic acid-enhanced MRI. For each observer, diagnostic accuracy (area under the alternative free-response receiver operating characteristic curve (Az)) was calculated and compared.

The index of diagnostic performance was determined as the sum of lesions correctly rated as definitely benign (1) or preferentially benign (2) among benign lesions and as preferentially malignant (4) or definitely malignant (5) among malignant lesions.

## Results

**Figure 2:**
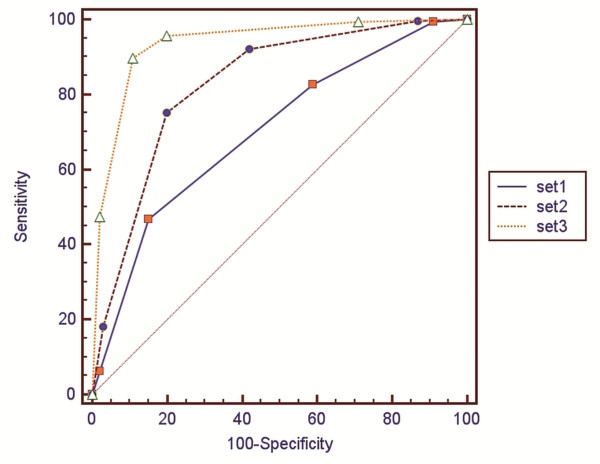
**ROC analysis of each set for all observers.** Areas under the curve were 0.708 for set 1, 0.833 for set 2, and 0.941 for set 3.

Az values for each observer are shown in Table 2. For all 5 observers, Az values for combined unenhanced and gadoxetic acid-enhanced dynamic and hepatobiliary phase images (set 3; observer 1: 0.890; observer 2: 0.923; observer 3: 0.934; observer 4: 0.975; observer 5: 0.984 were significantly higher than those for unenhanced images (set 1; observer 1: 0.677 (P = 0.002); observer 2: 0.695 (P = 0.002); observer 3: 0.634 (P < 0.0001); observer 4: 0.725 (P = 0.0001); observer 5: 0.810 (P = 0.002)). Four out of the 5 observers achieved higher Az values for combined unenhanced and gadoxetic acid-enhanced dynamic images (set 2; observer 1: 0.659; observer 2: 0.837, observer 3: 0.789; observer 4: 0.892; observer 5: 0.989) than for unenhanced images (set 1); for one observer, values were not statistically significant. Furthermore, 4 out of 5 observers got higher Az values for additional hepatobiliary phase images, and 3 out of these 4 observers achieved a significant increase (observer 1: P = 0.001; observer 3: P = 0.009; observer 4: P = 0.004;). The mean Az value of all observers in set 2 (mean Az = 0.833; 95% confidence interval (CI) = 0.788-0.863) was significantly higher than in set 1 (mean Az = 0.708; 95% CI = 0.655-0.745; P = 0.0002). The mean Az value of all observers in set 3 (mean Az = 0.941; 95% CI = 0.908-0.958) was significantly higher than in set 2 (P < 0.0001) (Figure 2 and Table 3).

**Table 2:** **A**_**z **_**values for characterization of focal solid hepatic lesions for each observer (Sets are defined in the Methods section)**

**Observer**	**Set 1**	**Set 2**	**Set 3**	**p value Set 1 vs. Set 2**	**p value Set 2 vs. Set 3**
Observer 1	0.677	0.659	0.890	0.835	0.001
Observer 2	0.695	0.837	0.923	0.112	0.081
Observer 3	0.634	0.789	0.934	0.047	0.009
Observer 4	0.725	0.892	0.975	0.014	0.004
Observer 5	0.810	0.989	0.984	0.0004	0.748

**Table 3:** **Diagnostic performance of all observers (mean A**_**z**_**) for imaging sets**

	**Set 1**	**Set 2**	**Set 3**	**p value set 1 vs. set 2**	**p value set 2 vs. set 3**
Mean A_Z_	0.708	0.833	0.941	0.0002	<0.0001
95% CI	0.655-0.745	0.788-0.863	0.908-0.958		

The index of diagnostic performance regarding the differentiation between benign and malignant lesions was 45% in unenhanced T1- and T2-weighted images (Set 1). Accuracy improved in unenhanced and gadoxetic acid-enhanced dynamic images (Set 2; 71%) and was highest in set 3 with additional hepatobiliary phase images (94%).

With unenhanced T1- and T2-weighted images (set 1), 37.9% of the lesions were rated as unclear (score 3). With dynamic phase images (set 2), this rate could be lowered to 18.1%. The combination of unenhanced images, dynamic images, and additional hepatobiliary phase images (set 3) further decreased this rate to 6.7%.

**Figure 3:**
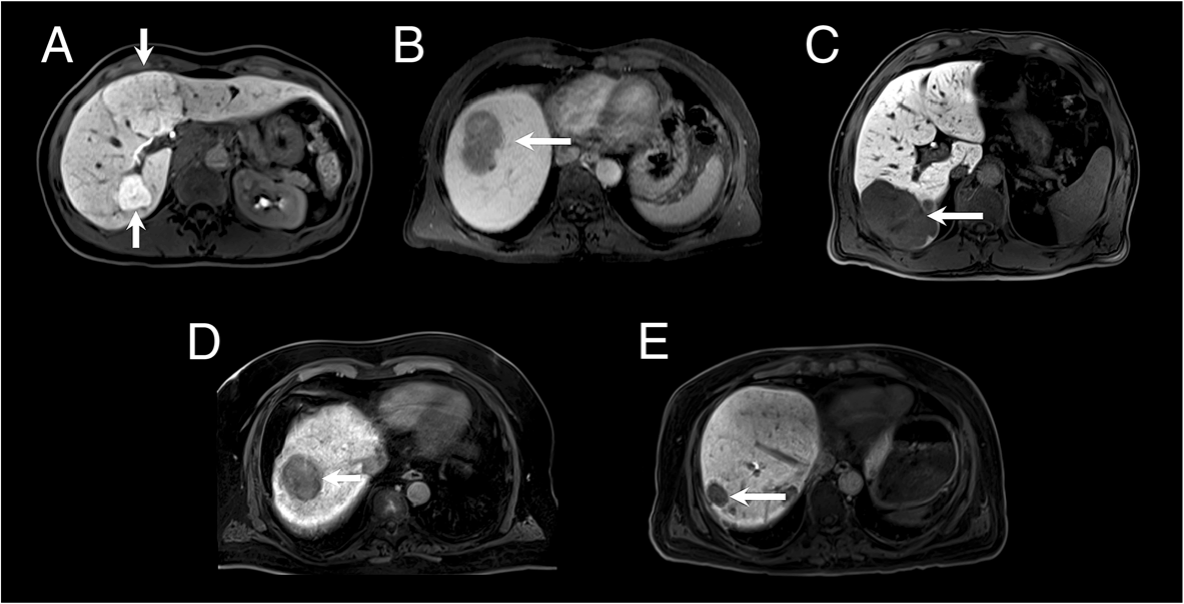
**Hepatobiliary phase images of benign and malignant focal solid hepatic lesions. A-E**, Transverse 3D fat-suppressed T1-weighted gradient-echo sequence (TR/TE: 4.0/1.5; flip angle: 10°; slice thickness: 6 mm) obtained 20 min after Gd-EOB-DTPA administration in hepatobiliary phase: white arrows depict **(A)** hyperintense FNHs with central scar, **(B)** hypointense adenoma, **(C)** hypointense hemangioma, **(D)** hypointense HCC and **(E)** hypointense metastasis.

A representative image of each benign and malignant focal solid hepatic lesion is shown in Figure 3. Imaging findings of respective benign and malignant lesions are shown in Table 4.

**Table 4:** Imaging findings at Gadoxetic acid-enhanced MRI

	**HCC (n = 34)**	**Metastasis (n = 30)**	**FNH (n = 14)**	**Hemangioma (n = 3)**	**Adenoma (n = 3)**
**Arterial phase imaging**					
Hyperintense	23	2	14	0	3
Isointense	8	12	0	0	0
Hypointense	3	16	0	3	0
**Portal venous phase imaging**					
Hyperintense	7	0	7	0	2
Isointense	12	9	7	0	1
Hypointense	15	21	0	3	0
**Equilibrium phase imaging**					
Hyperintense	5	0	7	0	2
Isointense	10	8	7	0	1
Hypointense	19	22	0	3	0
**Hepatobiliary phase imaging**					
Hyperintense	1	0	7	0	0
Isointense	7	3	6	1	0
Hypointense	26	27	1	2	3

## Discussion

The early detection and characterization of hepatic lesions has become increasingly important because surgical and minimally invasive interventional techniques may cure or at least improve diagnosis [16].

Several trials have proven that contrast-enhanced MRI is more sensitive in the detection of hepatic metastases and HCC than dynamic contrast-enhanced CT, probably because of the superior delineation of the contrast between lesion and liver and a more subtle depiction of the different tissue properties [3,11,17,18].

Therefore, numerous magnetic resonance contrast agents are available to facilitate the detection and characterization of hepatic lesions in MRI. Among these agents, Gd-EOB-DTPA has become increasingly more important because this contrast medium is able to create both dynamic phase images as well as liver specific (hepatocyte phase) MR images within the same examination [19].

In non-cirrhotic patients, the liver-specific hepatocyte phase can be scanned as early as 10 min after the injection of the contrast medium; however, many patients undergoing liver MRI suffer from liver cirrhosis that results in delayed Gd-EOB-DTPA hepatocyte uptake. Thus, a 20 min delay appears to be more appropriate [20]. Several trials have shown the best liver-to-lesion contrast 20 min after the injection of Gd-EOB-DTPA without any significant improvement in hepatobiliary phase images later than 20 min post-injectionem [12,21].

In early dynamic images, hypervascular benign hepatic lesions (e.g. hemangioma, FNH, adenoma) have to be distinguished from hypervascular metastases and HCCs that show arterial enhancement. Thus, if extracellular contrast agents are used, HCCs frequently appear isointense on equilibrium phase images, which might impede correct differentiation [22,23]. In the majority of cases in our trial, however, the use of gadoxetic acid-enhanced MR images led to rapid and strong enhancement of the liver parenchyma that showed arterial enhancement and clear wash-out during the late dynamic images, which increased diagnostic accuracy. However, enhancement patterns of HCCs on dynamic phase images and hepatobiliary phase images are dependent from type and progression of hepatocarcinogenesis. HCCs with atypical enhancement patterns in terms of missing arterial hypervascularity and consecutive missing wash-out pattern in delayed phase are shown in majority of cases to have a lower histologic grade, lower tumor aggressiveness, and a consecutive better clinical outcome [24]. However, atypical HCCs carry the risk to be overlooked in dynamic phase images and Gd-EOB-DTPA-enhanced hepatobiliary phase images have been shown to improve both detection and characterization of those lesions [15]. Only recently the malignant potential of hypointense lesions detected in the hepatobiliary phase of Gd-EOB-DTPA-enhanced MRI has been shown emphasizing its clinical benefit [25].

MRI is widely used for the differential diagnosis of benign hepatic lesions, such as FNH and hepatocellular adenoma (HCA). Both types of lesions are managed rather differently: whereas FNH do usually not require resection and are treated conservatively, HCA are commonly surgically removed to avoid serious clinical consequences [26]. The two benign tumors appear as hypervascular lesions on enhanced dynamic images, which leads to diagnostic problems in differential diagnosis. However, it could be shown recently that Gd-EOB-DTPA-enhanced hepatobiliary phase MRI facilitates the accurate differentiation between FNH and HCA: whereas FNH lesions appeared hyperintens in comparison to the adjacent liver parenchyma, adenoma were markedly hypointens in hepatobiliary phase images [27].

Furthermore, other trials have shown that Gd-EOB-DTPA-enhanced hepatobiliary phase MRI is superior to CT or standard gadolinium chelates, particularly in the differential diagnosis of hypervascular lesions and in the detection of small metastases, because detection rates and correct characterization have increased [8,19,28].

The results of our trial show the significant advantage of Gd-EOB-DTPA-enhanced MRI in evaluating hepatic tumor specimens:

The diagnostic performance of 4 out of 5 observers was better with early dynamic phase images than with plain T1- and T2-weighted images, whereas the diagnostic performance of 1 observer did not improve after the addition of dynamic images (Az set 1: 0.677; Az set 2: 0.659).

However, the additional information on tissue structure gained from Gd-EOB-DTPA-enhanced MRI during the hepatobiliary phase is of high importance for the interpretation of focal solid hepatic lesions, thus this tool still aids diagnostic accuracy. In our trial, the Az value could be increased with additional hepatobiliary phase images for 4 out of 5 observers. However, the observer whose performance did not improve with hepatobiliary phase images (observer 5) had already shown the highest diagnostic performance with Gd-EOB-DTPA-enhanced dynamic images (Az Set 2: 0.989). Regarding the fact that observer 5 had 16 years of experience in abdominal imaging and observer 1 was the most inexperienced of the 5 observers, the added value of Gd-EOB-DTPA-enhanced hepatobiliary phase images seems to be most apparent for radiologists who are not only specialized in abdominal MRI. Therefore, Gd-EOB-DTPA-enhanced dynamic imaging might be a crucial tool for quotidian use in a university hospital, where most radiologists are expected to run several imaging-units simultaneously.

The index of diagnostic performance showing a mean overall accuracy of the respective set for all 5 observers was significantly higher with dynamic images (71%) compared to T1- and T2- weighted images (45%) and highest with the addition of hepatobiliary phase images in set 3 (94%). This increase was very likely caused by the constant decrease of score 3, meaning “unclear” in subsequent sets ranging from 38% in set 1 to 7% in set 3.

Our trial has several limitations. First, we did not conduct a needle biopsy of every hepatic nodule. The majority of malignant lesions was either proven by histology or based on surgical findings or on results of percutaneous biopsy. There was only a low number of benign lesions and benign lesions were not histologically confirmed but regarded as benign if they remained unchanged at the follow-up examinations for more than 6 months without treatment in combination with typical imaging findings in previous examinations. Second, Gd-EOB-DTPA-enhanced MRI was conducted regardless of the liver function; delayed hepatocyte uptake concurs with diminished liver function, which might have influenced the diagnostic performance, particularly in case of HCC on cirrhosis. Consequently, it may be difficult to clearly define the value of contrast-enhanced dynamic and hepatobiliary phase images.

## Conclusion

The results of our trial support the conclusion that Gd-EOB-DTPA contrast-enhanced hepatobiliary phase MRI has an additional diagnostic value for the differential diagnosis of focal solid hepatic lesions in clinical routine and therefore the potential to improve diagnostic performance; notably, the added diagnostic value of Gd-EOB-DTPA-enhanced hepatobiliary phase MRI became more apparent particularly for radiologists with less experience in liver MRI.

In summary, Gd-EOB-DTPA-enhanced MRI is a useful tool for assessing the possible malignancy of focal solid hepatic lesions in clinical routine and therefore for the staging of malignant disease prior to surgery or percutaneous interventional therapies.

## Abbreviations

AFP: α-fetoprotein; Az: Area under the alternative free-response receiver operating characteristic curve; CT: Computed tomography; FNH: Focal nodular hyperplasia; HCA: Hepatocellular adenoma; HCC: Hepatocellular carcinoma; MRI: Magnetic resonance imaging; US: Ultrasonography.

## Competing interests

The authors declare that they have no competing interest.

## Authors’ contribution

MH performed the statistical analysis, did the literature search, interpretated data and drafted the manuscript. MW and IP participated in its design, collected the data and edited the manuscript. RM and CN helped in data acquisition, literature search and interpretation of data. PH and AS revised the manuscript critically for important intellectual content and made substantial contributions to data analysis. CS and PW participated in its design, coordination and statistical analysis and helped to draft the manuscript. All authors read and approved the final manuscript.

## Pre-publication history

The pre-publication history for this paper can be accessed here:

http://www.biomedcentral.com/1471-2342/13/41/prepub
